# Helminth infections of great concern among cattle in Nigeria: Insight to its prevalence, species diversity, patterns of infections and risk factors

**DOI:** 10.14202/vetworld.2020.338-344

**Published:** 2020-02-21

**Authors:** Shola David Ola-Fadunsin, Isau Aremu Ganiyu, Musa Rabiu, Karimat Hussain, Idiat Modupe Sanda, Alhassan Yunusa Baba, Nathan Ahmadu Furo, Rashidat Bolanle Balogun

**Affiliations:** 1 Department of Veterinary Parasitology and Entomology, Faculty of Veterinary Medicine, University of Ilorin, P.M.B. 1515 Ilorin, Kwara, Nigeria; 2 Department of Veterinary Medicine, Faculty of Veterinary Medicine, University of Ilorin, P.M.B. 1515 Ilorin, Kwara, Nigeria; 3 Veterinary Teaching Hospital, University of Ilorin, P.M.B. 1515, Ilorin, Kwara, Nigeria

**Keywords:** cattle, helminths, Nigeria, prevalence, risk factors

## Abstract

**Background and Aim:**

Helminth infections are one of the greatest causes of productive and reproductive loss in animals and man, and in some cases, it results in heavy mortalities. This study was conducted to determine the prevalence, species diversity, patterns of infections and risk factors associated with helminth infections of cattle in Ilorin, Nigeria.

**Materials and Methods:**

A total of 478 fecal samples were collected from abattoirs and cattle farms over a year period (March, 2018-February, 2019). Fecal samples were visually examined then observed using simple flotation and formalin-ethyl acetate sedimentation techniques. Eggs and worms were identified according to standard procedures. The packed cell volume was determined using the hematocrit centrifugation technique.

**Results:**

A total of 79.92% of the cattle examined were found positive with one or more helminth species. Eighteen helminth species (cutting across all classes of helminths) were detected, with *Haemonchus contortus* (60.46%), *Trichostrongylus* spp. (46.44%), *Ostertagia ostertagi* (42.05%), *Bunostomum phlebotomum* (28.87%), *Cooperia* spp. (24.27%), *Oesophagostomum radiatum* (21.97%), *Strongyloides papillosus* (12.13%), and *Fasciola gigantica* (10.67%) been the most prevalent. Helminth infection was detected all through the year with the least prevalence recorded in February (55.00%). About 61% of the examined cattle harbored double/multiple helminth species. There was a significant difference between breed, sex, physiological status, and season with the prevalence rate of helminth infections (p<0.05).

**Conclusion:**

Our investigation demonstrated high prevalence and wide diversity of helminth species, which suggests that helminth infections are of great concern among cattle in Ilorin and Nigeria in general. There is a need for a radical veterinary intervention to curb the menace so as to have an economically robust cattle industry in Nigeria.

## Introduction

Livestock (including cattle) farming is among the major sectors representing a valuable asset in both traditional and modern agriculture in sub-Saharan Africa, as well as in other tropical and subtropical regions of the world providing animal protein, milk, and beef during festivities around the world, flexible income for family units, employment, hides and skin for leather production, farm energy, and manure [1,2].

Nigeria is the largest livestock producer in sub-Saharan Africa, with a population of about 17 million cattle, and a larger number of this population is concentrated in the northern region of the country [3,4]. The worth of Nigeria’s livestock is estimated to the tone of USD 6 billion [5] and they contribute greatly to the agricultural component of the gross domestic product (GDP) of which cattle production makes up to 40% [5,6]. Despite the nation’s indigenous population, a good number of cattle are imported from neighboring African countries to meet the demands of meat in major cities in Nigeria [7].

Helminths of ruminants refer to a group of complex multicellular eukaryotic parasites which are infective to animals all over the world [8]. Helminth infection is a menace for both small- and large-scale farmers, but their impact is greater in sub-Saharan Africa including Nigeria, due to the availability of a wide range of agro-ecological factors suitable for diversified hosts and parasite species [9]. Economic losses associated with helminth parasitic conditions range from decreased utilization of feeds in unthrifty animals to weight loss or even death [10].

Helminth infection is one of the major causes of wastage and decreased productivity exerting their effect through mortality, morbidity, decreased growth rate, weight loss in young growing calves, and late maturity of slaughter stock. It also causes unthriftiness, gut damage, anemia, diarrhea, anorexia, gastroenteritis, abdominal distention, emaciation, reduced feed intake, and reduced absorption of nutrients, reduced milk and meat production, and working capacity of the animal mainly in developing countries [6,11].

The epidemiology of parasite infections in cattle has been well documented in several countries, which helped improve helminths control, animal performance, and decrease in production losses [2,11,12].

There is a dearth of information about helminths of cattle in Nigeria, as this study appears to be the first on the subject matter in Ilorin, North-Central Nigeria. In this study, we determined the prevalence, species diversity, dynamics of infections and risk factors associated with helminth infections among cattle in Ilorin, Nigeria. The findings from this study will enrich the database of internal helminths of cattle and disease surveillance which is extremely important in the management of cattle to achieve improved production.

## Materials and Methods

### Ethical approval

All applicable international, national, and/or institutional guidelines for the collection of fecal and blood samples from cattle were appropriately followed.

### Study area

**Figure-1:**
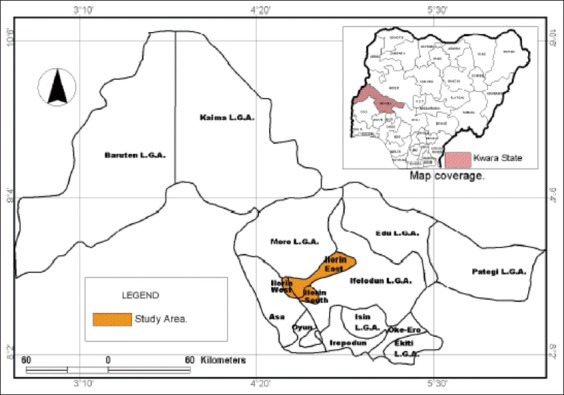
Map of Kwara State showing the location of Ilorin metropolis (study area). Insert map showing the location of Kwara State in Nigeria [13].

The study was carried out in Ilorin metropolis (which comprises Ilorin West, Ilorin East, and Ilorin South Local Government Areas). Ilorin is situated almost at the middle of Nigeria, and hence, it is popularly referred to as the “connecting city of Nigeria” (Figure-1). Ilorin is the administrative capital of Kwara State, and the state is located in the North-Central part of Nigeria within the forest-savanna region. Kwara State is located within latitude 8° 30′N and longitude 5° 00′E and covers an area of 35,705 km^2^ (13,947.27 Sq. miles). With regard to the climate, the state has two major seasons, the dry (December to March and August) and wet (April to July and September to November) seasons, with heavier rainfall in September and October. The state has a mean annual rainfall of between 112.8 cm and 146.9 cm and an average annual temperature ranging from 22.1°C to 33.3°C. It records a mean relative humidity of 49.6% [3,13,14].

### Study population and sampling

The study was conducted from March 2018 to February 2019. A total of 478 cattle were sampled monthly from abattoirs and different cattle herds. A random sampling technique was used to select cattle for the study. The age of the sampled cattle was estimated, as described by Lasisi *et al*. [15]. Body condition scores (BCS) were recorded using the protocol as described by Shittu *et al*. [16]. About 5 g of fecal samples were collected from the rectum directly into clean polyethylene disposal bags and the collected samples were labeled accordingly. Furthermore, about 5 ml of blood were collected either at slaughter (from slaughtered cattle) or through the jugular or coccygeal venipuncture using an 18-gauge needle for adult cattle and 20-gauge needle for calves (from cattle in farms). The blood samples were collected into a labeled ethylenediaminetetraacetic acid tube. The samples (blood and fecal) were then put in separate cool boxes and were immediately transported to the Parasitology Laboratory of the Faculty of Veterinary Medicine, University of Ilorin, Nigeria, for further parasitological processing. Fecal consistency was assessed immediately after sampling and classified as normal or diarrheal without any additional differentiation [17].

### Coprological examination and identification of helminth eggs and adult helminths

Parasitological examinations were carried out using standard methods. Fecal samples were visually observed for adult helminths and then examined by means of the simple flotation (using saturated NaCl solution) and the formalin-ethyl acetate sedimentation techniques, as described by Ola-Fadunsin *et al*. [18]. Adult helminths were examined with the aid of a hand lens. Identification of helminth eggs and adult helminths was based on helminthological keys, as described by Soulsby [19], Foreyt [20], and Taylor *et al*. [21].

### Determination of packed cell volume (PCV)

PCV was determined using the hematocrit centrifugation technique, as described by Cheesbrough [22]. Briefly, capillary tubes were filled with blood by means of capillary forces. The filled capillary tubes were sealed with sealant. The sealed capillary tubes were centrifuged at 11,800 rpm for 5 min. The PCV was then read using a microhematocrit card reader. The PCV was categorized into anemic (≤30%) and nonanemic (>30%), as described by Fielder [23].

### Determination of positivity

Fecal samples that were positive following one or more of the examination techniques carried out (the simple flotation technique, the formalin-ethyl acetate sedimentation technique nor adult helminths observation) were considered positive.

### Statistical analysis

All data collected from the study were recorded in a Microsoft Excel spreadsheet and worked on therein. Statistical analyses were carried out using the Statistical Package for the Social Sciences (SPSS, Chicago, Illinois, USA) for Windows version 22.0. Descriptive statistics were conducted to estimate the prevalence using percentages in tables. The prevalence was calculated as the ratio between the number of cattle having helminth egg(s)/adult helminth(s) and the total number of sampled cattle. The univariate analysis (Chi-square) test and odds ratios (ORs) with 95% confidence interval (CI) were used to determine the association between each risk factor and the presence or absence of adult helminth(s) and helminth egg(s). The ORs were calculated with respect to a reference category, as indicated in the respective tables. The values were statistically different when p<0.05.

## Results

### Total prevalence (%) and diversity of helminth species

Of the total number of cattle sampled, 79.92% (382/478) were infected with one or more helminth species. Eighteen different helminth species comprising 12 gastrointestinal nematodes, four trematodes, and two cestodes were detected with *Haemonchus contortus* (289/478; 60.46%) been the most prevalent helminth species overall. *Trichuris* spp. (3/478; 0.63%) was the least prevalent overall. Of the phylum Nematoda, the prevalence of 46.44%, 42.05%, and 5.86% was detected for *Trichostrongylus* spp., *Ostertagia ostertagi*, and *Toxocara vitulorum*, respectively. *Fasciola gigantica* and *Moniezia benedeni* were the most prevalent in the classes *Trematoda* and *Cestoda*, respectively (Table-1).

**Table-1:** Total prevalence (%) of helminth species among cattle in Ilorin, Nigeria.

Helminth species	Number infected	Prevalence (%)	95% confidence interval
Nematodes			
*Haemonchus contortus*	289	60.46	56.02-64.77
*Trichostrongylus* spp.	222	46.44	42.00-50.93
*Ostertagia ostertagi*	201	42.05	37.68-46.52
*Bunostomum phlebotomum*	138	28.87	24.94-33.06
*Cooperia* spp.	116	24.27	20.58-28.26
*Oesophagostomum radiatum*	105	21.97	18.43-25.85
*Strongyloides papillosus*	58	12.13	9.43-15.29
*Chabertia ovina*	32	6.69	4.70-9.21
*Toxocara vitulorum*	28	5.86	4.01-8.24
*Gongylonema pulchrum*	4	0.84	0.27-2.01
*Nematodirus* spp.	4	0.84	0.27-2.01
*Trichuris* spp.	3	0.63	0.16-1.70
Trematodes			
*Fasciola gigantica*	51	10.67	8.13-13.68
*Fasciola hepatica*	31	6.49	4.53-8.97
*Paramphistomum cervi*	26	5.44	3.66-7.76
*Dicrocoelium dendriticum*	12	2.51	1.37-4.23
Cestodes			
*Moniezia benedeni*	12	2.51	1.37-4.23
*Moniezia expansa*	6	1.26	0.51-2.59

### Monthly prevalence (%) of helminth species

**Figure-2:**
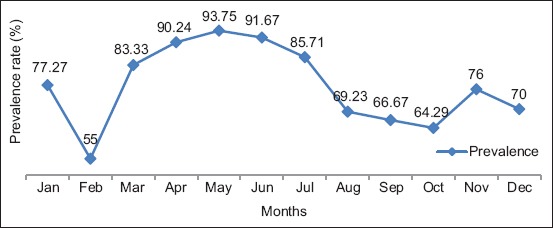
Monthly prevalence (%) of helminth species among cattle in Ilorin, Nigeria.

The monthly prevalence of helminth species is presented in Figure-2. There was no defined pattern in the monthly prevalence of parasitic helminths during the 1 year of study, although a high prevalence of 83.33%-85.71% was recorded between March and July. The least prevalence as recorded in February (55.00%).

### Patterns of helminth infections

Of the 478 cattle sampled, 20.08% (96/478) were free from helminth infections. The prevalence pattern, almost perfectly decreased as the patterns of infections increased. Two, three, four, and five helminth coinfections recorded a prevalence of 13.39%, 12.13%, 8.58%, and 10.88%, respectively (Table-2).

**Table-2:** Patterns of helminth infections among cattle in Ilorin, Nigeria (n=478).

Patterns of infections	Number infected	Prevalence (%)	95% confidence interval
0	96	20.08	16.67-23.86
1	91	19.04	15.71-22.74
2	64	13.39	10.55-16.67
3	58	12.13	9.43-15.29
4	41	8.58	6.31-11.35
5	52	10.88	8.32-13.91
6	39	8.16	5.95-10.88
7	15	3.14	1.84-5.01
8	16	3.35	1.99-5.27
9	6	1.26	0.51-2.59

### Risk factors associated with helminth infections

In general, breed was not significantly associated with the prevalence of helminth infections among cattle (*χ*^2^=5.79) although, the Sokoto Gudali breed was 2.3 times more likely to be infected with helminth species than the White Fulani and the difference was significant (p=0.03). Male was about 6.3 times more likely to be infected with helminths compared to female (*χ*^2^=8.42; p<0.01; OR=0.16; 95% CI=0.03-0.55). Physiological status was significantly associated with the prevalence of helminths (*χ*^2^=5.79; p=0.02). Higher prevalence (OR=2.35; 95 % CI=1.01-6.25) was recorded in the young compared to physiologically dry cattle. Helminth infections were 2 times more likely to occur during the wet season than the dry season and this difference was significant (p<0.01). Age, BCS, fecal consistency, and PCV level were not significantly associated with the prevalence of helminth infections among cattle (Table-3).

**Table-3:** Prevalence and epidemiological variables that were investigated as potential risk factors for helminth infections among cattle in Ilorin, Nigeria.

Variable	Positive (%)	Negative (%)	OR (95% CI)	p	χ^2^ value
Breed					
Red Bororo	80 (76.92)	24 (23.08)	0.93 (0.53-1.64)	0.79	5.79
Sokoto Gudali	66 (89.19)	8 (10.81)	2.30 (1.06-5.45)	0.03^†^	
Kuri	12 (75.00)	4 (25.00)	0.84 (0.27-3.13)	0.75	
Keteku	38 (79.17)	10 (20.83)	1.06 (0.50-2.38)	0.90	
Friesian cross	14 (87.50)	2 (12.50)	1.95 (0.48-13.05)	0.41	
White Fulani*	172 (78.18)	48 (21.82)	1.00		
Age (years)					
≤1	40 (100.00)	0 (0.00)	X	X	14.13
>1-≤4	143 (82.18)	31 (17.82)	1.41 (0.75-2.61)	0.28	
>4-≤10	127 (74.71)	43 (25.29)	0.90 (0.49-1.62)	0.74	
>10*	72 (76.60)	22 (23.40)	1.00		
Sex					
Female	336 (78.14)	94 (21.86)	0.16 (0.03-0.55)	<0.01^†^	8.42
Male*	46 (95.83)	2 (4.17)	1.00		
Body condition score					
Emaciated	81 (78.64)	22 (21.36)	0.99 (0.53-1.87)	0.97	0.41
Moderate	193 (81.09)	45 (18.91)	1.15 (0.68-1.94)	0.60	
Good*	108 (78.83)	29 (21.17)	1.00		
Physiological status					
Young	51 (89.47)	6 (10.53)	2.35 (1.01-6.25)	0.04^†^	12.23
Mating stock	16 (100.00)	0 (0.00)	X	X	
Pregnant	6 (54.55)	5 (45.45)	0.33 (0.09-1.22)	0.09	
Lactating	63 (78.75)	17 (21.25)	1.02 (0.57-1.91)	0.95	
Dry*	246 (78.34)	68 (21.66)	1.00		
Fecal consistency					
Soft	178 (80.54)	43 (19.46)	1.08 (0.69-1.69)	0.75	0.10
Normal*	204 (79.38)	53 (20.62)	1.00		
PCV					
≤30%	157 (83.07)	32 (16.93)	1.40 (0.87-2.25)	0.17	1.94
>30%*	225 (77.85)	64 (22.15)	1.00		
Season					
Dry	138 (72.63)	52 (27.37)	0.48 (0.30-0.75)	<0.01^†^	10.43
Wet*	244 (84.72)	44 (15.28)	1.00		

OR=Odds ratio, CI=Confidence interval.

* Reference category.

† Significant. PCV=Packed cell volume

## Discussion

Helminth infections in ruminants and cattle, in particular, are recognized as a major constraint to livestock production. In most cases, infections are subclinical with significant economic losses due to mortality, reduced productivity, and reproductivity of animals [12]. This present body of evidence revealed that 79.92% of the sampled cattle in Ilorin (a central city in Nigeria) were plagued with one or more parasitic helminth species. A total of 18 different species of helminths were detected to infect cattle in the study. The prevalence of parasitic helminths in cattle has been reported ranging from 4.30% to 87.41% in Nigeria [7,24] and 32.20% to 78.02% in other parts of the world [25,26]. The variations in our finding with other reports might be due to the difference in the sample size, selection of samples, breed(s) of cattle, climatic conditions, management practices, grazing habits of the cattle, level of education of the farmers, the availability of intermediate hosts, the laboratory technique(s) conducted and the period, duration, and place of study [12,27].

Eighteen different helminth species were detected in this study. In Nigeria, researches such as Yahaya and Tyav [1], Okike-Osisiogu *et al*. [24], Karaye *et al*. [28], Lemy and Egwunyenga [4], Nnabuife *et al*. [29], and Yuguda *et al*. [10] had reported four, six, nine, ten, eleven and thirteen different helminth species, respectively. Outside Nigeria, helminth species diversity of four to nine has been reported [11,30]. The high prevalence and diversity of helminth species recorded in this study indicate that helminth infections are of great concern among cattle in the study area and Nigeria in general.

Gastrointestinal nematodes were the most detected helminth class, followed by trematodes and cestodes. Nematodes have been documented to be the most numerous class of helminths infecting cattle worldwide [2,9,11,29,30]. *Haemonchus contortus*, *Trichostrongylus* spp., *O. ostertagi*, and *Bunostomum phlebotomum* were the most prevalent helminth species in this study. Similarly, Lemy and Egwunyenga [4], Okike-Osisiogu *et al*. [24], Abah and Ebong [31], Takeet *et al*. [32], and Njonge [33] reported the same in their respective studies. *Ostertagia ostertagi* is believed to be an important helminth of the temperate region [21], but studies in the tropical regions showed that the helminth is present and is of noticeable prevalence among cattle in the region [4,10]. The high prevalence of these gastrointestinal nematodes in this study may be associated with the direct life cycle of the helminths where no intermediate host is needed.

*Fasciola* species were the most prevalent trematode species in this study, with *F. gigantica* been more prevalent. *Fasciola* species has been reported to be the most prevalent trematode of cattle in Nigeria, with *F. gigantica* been more prevalent among the two species [4,10]. *Paramphistomum cervi* and *Dicrocoelium dendriticum* have also been detected among cattle in Nigeria [4,10,28]. *Fasciola gigantica* is the larger *Fasciola* species of cattle and it is commonly found in Africa than *Fasciola hepatica* [21].

*Moniezia benedeni* and *Moniezia expansa* were the only cestodes recorded in this study, with *M*. *benedeni* been more prevalent. These two helminths were the only cestodes detected in cattle in studies conducted in Ethiopia [11], India [26], and Columbia [34]. These cestodes have been reported to be common and of importance among cattle in Nigeria [10,24,28,29]. *Moniezia benedeni* is known to affect only cattle, while *M*. *expansa* is found in sheep, goats, and occasionally cattle [21]. *Moniezia benedeni* was documented to be the only cestode affecting cattle in a study conducted in Bejaia Province of Algeria [12]. Considering our findings with those reported from other parts of the world, it suggests that *Moniezia* species are the most prevalent and important cestodes of cattle, while *M*. *benedeni* been more important among the two.

Helminths (nematodes) with free-living stages can survive on pasture throughout the year [35], making infections possible at every month of the year. *Trichostrongylus* spp. has the ability to withstand more hostile seasons which facilitates both survival and reinfection all year round [36]. To these, we observed that helminth species were recorded in a noticeable prevalence among cattle throughout the year. Helminth infections have been detected all through the year among cattle in earlier studies [1,28].

In line with our finding, double and multiple parasitic helminth infections are a common phenomenon in cattle all over the world [1,25-27,33]. This may be associated with the grazing preference/nature of cattle, favorable climatic conditions, and the rate of environmental contamination with viable worm eggs at a particular time, which to a great extent can determine the establishment of mixed infections.

Breed is an important index in the epidemiology of helminth infections in cattle [10,33], with exotic and cross breeds believed to be more at risk to helminth infections compared to indigenous breeds [2]. Studies have also shown that indigenous cattle breeds are more at risk of helminth infections than the cross and exotic breeds [37]. In like manner, we reported a higher prevalence of helminth infection in the crossbreed (Friesian cross) and indigenous breed (Sokoto Gudali). Similarly, Ola-Fadunsin [6] reported that the Sokoto Gudali breed was most susceptible to helminth infections compared to other cattle breeds. The prevalence of helminth infections in relation to different cattle breeds is multifactorial: The type of management system the cattle is raised, the frequency of and type of anthelmintic used, and the physiological and nutritional status of the cattle.

The higher prevalence of helminth infections recorded in males compared to females may be attributed to the aggressive nature of male animals (cattle) when feeding, as this may cause them to pick up more helminth eggs on the pasture, making them more susceptible to helminthosis [6]. Furthermore, male domestic ungulates are said to be more susceptible to infections with gastrointestinal tract parasites than females due to hormones debilitating immune functions, which favor the growth and spread of parasites in male guts [6,38]. A higher prevalence of helminth infections has been reported in male cattle compared to females in different parts of the world [1,2,11,24,39].

The physiological status of animals (including cattle) is a significant risk factor associated with helminth infections [40]. We observed the highest prevalence of helminth infection in young and mating stock compared to the other groups in the category. Ola-Fadunsin [6] and Njonge [33] reported a higher prevalence of helminth infections in calves compared to adult cattle. The naiveness of the immune system of calves and the stress associated with hormonal (testosterone and estrogen) interplay in matting stock may be responsible for the high prevalence recorded in these groups of cattle.

Helminth infections were more common during the wet season than the dry season in our study. This is not novel as researchers elsewhere in the world has reported similar findings [1,2,36,41,42]. Our observation may be attributed to the high moisture content and lower temperature which favors the growth and development of helminth eggs and larvae on pasture, leading to infections in cattle.

All cattle were infected with helminths with no bias with respect to age, BCS, fecal consistency, and the PCV of the cattle. In a related manner, Bisimwa *et al*. [2] observed no age disparity in the prevalence of parasitic helminths among cattle in Congo. Furthermore, Kabaka *et al*. [39] documented that there was no significant association between helminth infections in cattle and BCS in their study conducted in Kenya. Not all parasitic helminths of cattle are associated with diarrhea and anemia [21], as such, it is not surprising those helminth infections in cattle is not significantly associated with fecal consistency and PCV levels.

## Conclusion

To the best of our knowledge, this is the first report on helminths of cattle in Ilorin, North-Central Nigeria. Our study demonstrated a high prevalence and wide helminth species diversity, suggesting that helminth infections are of great concern among cattle in Ilorin and Nigeria in general. All classes of helminths were detected in this study, with nematodes been the most prevalent and numerous. Over three-fifths of the studied cattle harbored double/multiple helminth species. There was a relationship between breed, sex, physiological status and season, and the distribution of helminth infections in cattle. There is a great need for periodic and strategic use of anthelmintics so as to curb this scourge, as helminthosis causes great economic losses to both beef and dairy farmers and the nation’s GDP.

## Authors’ Contributions

SDO conceived and designed the research work, was involved in sample collection and laboratory analysis. He also did the data analysis and drafted the manuscript. IAG and MR were involved in sampling and in the laboratory work. KH and IMS were involved in the laboratory analysis. AYB, NAF, and RBB partook in the sample collection. All authors read and ratified the final manuscript.
